# Multi-Stage Feature Extraction and Classification for Ship-Radiated Noise

**DOI:** 10.3390/s22010112

**Published:** 2021-12-24

**Authors:** Hamada Esmaiel, Dongri Xie, Zeyad A. H. Qasem, Haixin Sun, Jie Qi, Junfeng Wang

**Affiliations:** 1 Department of Information and Communication, School of Informatics, Xiamen University, Xiamen 316005, China; h.esmaiel@aswu.edu.eg (H.E.); zeyadqasem@stu.xmu.edu.cn (Z.A.H.Q.); 2 Electrical Engineering Department, Faculty of Engineering, Aswan University, Aswan 81542, Egypt; 3 China Electronics Technology Avionics Co., Ltd., Chengdu 610100, China; 23320171153152@stu.xmu.edu.cn; 4 School of Electronic Science and Engineering, Xiamen University, Xiamen 361005, China; qijie@xmu.edu.cn; 5 Department of Information and Communication Engineering, School of Electrical and Electronic Engineering, Tianjin University of Technology, Tianjin 300383, China; great_seal@163.com

**Keywords:** ship-radiated noise, variational mode decomposition, weighted permutation entropy, local tangent space alignment

## Abstract

Due to the complexity and unique features of the hydroacoustic channel, ship-radiated noise (SRN) detected using a passive sonar tends mostly to distort. SRN feature extraction has been proposed to improve the detected passive sonar signal. Unfortunately, the current methods used in SRN feature extraction have many shortcomings. Considering this, in this paper we propose a new multi-stage feature extraction approach to enhance the current SRN feature extractions based on enhanced variational mode decomposition (EVMD), weighted permutation entropy (WPE), local tangent space alignment (LTSA), and particle swarm optimization-based support vector machine (PSO-SVM). In the proposed method, first, we enhance the decomposition operation of the conventional VMD by decomposing the SRN signal into a finite group of intrinsic mode functions (IMFs) and then calculate the WPE of each IMF. Then, the high-dimensional features obtained are reduced to two-dimensional ones by using the LTSA method. Finally, the feature vectors are fed into the PSO-SVM multi-class classifier to realize the classification of different types of SRN sample. The simulation and experimental results demonstrate that the recognition rate of the proposed method overcomes the conventional SRN feature extraction methods, and it has a recognition rate of up to 96.6667%.

## 1. Introduction

Ships are playing an increasingly important role in many military and civilian applications. For example, in military field applications, an effective prediction for enemy ships helps us to take the correct action and activate our countermeasure to avoid enemy attacks and defeat them. For civilian applications, a logical comprehensive analysis of different port noise, including ship-radiated noise (SRN) can help researchers support the reproduction of marine life [1]. For improving the passive sonar operation in ship applications, SNR feature extraction has been proposed [1]. However, marine environmental diversity provides a rich noise environment, and that increases the difficulty of extracting features reflecting the intrinsic characteristics of the ships [1]. In recent years, studies of the SRN feature extraction have increased. Unfortunately, the current SRN feature extraction schemes have many drawbacks. For example, Fourier transform (FT) [1] is only useful in only estimating the signal spectral information, but it is unsuccessful at time-varying representation. To address this drawback, short-time Fourier transform (STFT) has been proposed to indicate the time-varying signal traits. However, a fixed STFT window width makes STFT unable to consider good representation for the time domain and frequency domain at the same time [1]. To overcome the STFT drawbacks wavelet transform (WT) has been proposed by using the unfixed length of the window) [2]. The time-frequency decomposition is improved based on WT, but the wavelet basis function and decomposition layers are required to be set in advance, which practically limits the further application for WT in practice.

Empirical mode decomposition (EMD) has been proposed in [3], to decompose the signal into a group of limited intrinsic mode functions (IMFs), the core of the Hilbert transform (HT). The main purpose of the HT is to snaffle the component of the instantaneous frequency. Therefore, EMD can carefully and rapidly describe the instantaneous frequency of multi-components, hence EMD is more suitable for analyzing non-linear and non-stationary signals. However, the main EMD drawback is the mode mixing problem and many researchers have tried hard to address this issue. To this end, the ensemble empirical mode decomposition (EEMD) [4] has been offered as an amended EMD method [4]. EEMD has been proposed to solve the mode mixing problem by adding Gaussian white noise to the construed signal, and at the end averaging the obtained multiple decompositions results to obtain the IMFs. However, EEMD introduces and has additional problems, firstly, as, in the EMD, the EEMD decomposition results include residual components. Secondly, due to the randomness of Gaussian white noise, the outcome of EEMD is diverse between each decomposition time. Hence, the EEMD lacks a solid mathematical foundation to be distributed and widely accepted. Unlike the EMD and EEMD, variational mode decomposition (VMD) has been proposed in [5], which assumes that each mode is concerning a central frequency with restricted bandwidth. Thus, to obtain the center frequency and bandwidth of each component, VMD constantly searches for the modes and center frequency of each mode by using an alternating direction method of a multiplier, thereby solving the variational problem. In recent years, many works tried to extend the current EMD, EEMD, and VMD methods and applied these schemes in the fields of biomedical engineering [6,7,8,9], mechanical fault diagnosis [10,11,12], and acoustic signal processing [13,14]. In [13], the VMD is firstly performed to decompose the SRN signal, and the permutation entropy (PE) of each IMF with the highest energy is then extracted, achieving a recognition rate of 94%. In [14,15] the VMD decomposition of the SRN signal was performed and the fluctuation-based dispersion entropy (FDE) of each IMF was studied; the obtained IMF with the smallest difference from the FDE of the prime signal was then chosen to describe the raw signal with a recognition rate of 97.5%. In the EMD-EIMF-PE method proposed in [16], the signal-dominant IMF by EMD was chosen based on the energy gauge, and its PE was regarded as the feature parameter effectively distributing the SRN. Although both [13,14] have high recognition rates, there are still flaws in these methods. The mode VMD number has specified the EMD results in [13,14], which will certainly influence the VMD decomposition accuracy. Since the pertinent studies failed to strictly obtain the VMD model number, this paper suggests a new enhanced VMD (EVMD) method with the mode number close to the variance of IMFs’ center frequency.

Entropy is an important indicator to measure the uncertainty of time series and can consider the implicit system dynamics. When the system dynamics vary, the time series complexity will be varied as well. PE [15] has been used in mechanical fault diagnosis [16], agricultural commodity analysis [17], financial sequence analysis [18] due to its fast operation speed and excellent stability. Nevertheless, PE does not consider the case of neighboring vectors having the same ordinal patterns with different amplitudes, which will lead to the estimated value higher than the actual [19]. To this end, due to the preamble weights, the weighted permutation entropy (WPE) [20] is further critical to the amplitude-coded information in the signal and has outperformed the PE in combating the distortion caused by noise. To the best of the authors’ knowledge, WPE has been widely used in uncertainty measurements in many fields [21,22], but is rarely used in the SRN feature extraction.

With current computer technology development and the fast growth of data, it is of great necessity to further process the original high-dimensional data. Feature extraction can be divided into two parts; extracting the feature vectors can reflect the essence of analyzing signals through suitable signal processing methods. The other is selecting an appropriate measure decreases algorithm to minimize the increases, and in that way support the confession execution. Up to now, researchers have suggested different dimension relief algorithms, such as principal component analysis (PCA) [23,24], independent component analysis (ICA) [24], and linear discriminant analysis (LDA) [25]. PCA recognizes measurement saving by realizing optimal variance without dropping the creative data. The PCA and ICA are linear unsupervised dimension reduction algorithms. LDA is also a linear projection method that achieves dimension reduction by making the most of the ratio of the discrete matrix among classes and the discrete matrix within the class. Due to the SRN non-linear characteristics, linear dimension reduction algorithms are scarce in removing the intrinsic features of SRN signals. As a non-linear manifold learning algorithm, local tangent space alignment (LTSA) has been extensively useful in dimension reduction, thanks to its fast process speed and selfishness to selected parameters [26,27,28,29]. To the best of our knowledge, there is no study combining EVMD, WPE, and LTSA to classify underwater acoustic targets. Each method has its pros and cons, motivating us to combine all methods to have the maximum benefits.

To that end, this paper puts forward a novel multistage feature extraction method proposing an EVMD method and combining it with the WPE, and LTSA for SRN samples classification. In this paper, the proposed EVMD method uses the variance of the IMFs’ center frequency to calculate the mode number of VMD and enhance its operation. Next, the new EVMD algorithm is used to decompose the SRN signals plus calculate the WPE of each IMF. Then high-dimensional features are reduced to two-dimensional ones by the LTSA method. Finally, the feature vectors obtained are fed into the PSO-SVM multi-class classifier to recognize the different types of SRN samples.

The structure of the paper is presented as follows: the fundamental theories of the relevant algorithms are described in Section 2. In Section 3, the basic steps of the proposed method are presented. Section 4 applies the proposed method to the analysis of simulated signals. In Section 5, the proposed method is utilized for the feature extraction of SRN. Finally, the paper is concluded in Section 6.

## 2. Basic Theory

In this section, the theories of the related methods such as VMD, PE, WPE, and LTSA will be presented.

### 2.1. Variational Mode Decomposition (VMD)

The VMD defines the IMF in the function of the instantaneous amplitude $Am_{k}\left(t\right)$ and phase $Ph_{k}\left(t\right)$ as an amplitude-modulated-frequency-modulated (AM-FM) signal, given as below:

(1) $$I_{k}\left(t\right)=Am_{k}\left(t\right)\cos\left(Ph_{k}\left(t\right)\right)$$

where the change of the $Am_{k}\left(t\right)$ and ${\dot{Ph}}_{k}\left(t\right)$ are slower than $Ph_{k}\left(t\right)$. Each $I_{k}\left(t\right)$ is compacted around a respective center frequency with limited bandwidth obtained by Gaussian smoothing demodulation. In the VMD algorithm, decomposing the raw signal s(t) into a finite group of IMFs to find the variational problem can be expressed as follows:

(2) $$\underset{\left\{I_{k},f_{k}\right\}}{\underset{⏟}{\min}}\left\{\underset{k}{\sum }{||\partial }_{t}\left[\left(\delta \left(t\right)+\frac{j}{\pi t}\right)*I_{k}\left(t\right)\right]e^{-j2\pi f_{k}t}{\left|\right|}_{2}^{2}\right\},\;\;\;\;\;\;\;\;\;s.t.\underset{k}{\sum }I_{k}=s\left(t\right)$$

where $\partial_{t}$, δ(t) and $f_{k}$ represent the partial derivative, impulse function, and center frequency of $I_{k}\left(t\right)$, respectively. The constrained variation problem in Equation (2) is addressed using the quadratic penalty term and the Lagrange multipliers below:

(3) $$L\left(\left\{I_{k}\right\},\left\{f_{k}\right\},\lambda \right)=\alpha \underset{k}{\sum }{||\partial }_{t}\left[\left(\partial_{t}+\frac{j}{\pi t}\right)*I_{k}\left(t\right)\right]e^{-j2\pi f_{k}t}{\left|\right|}_{2}^{2}+\left||s(t\right)-\underset{k}{\sum }I_{k}\left(t\right){\left|\right|}_{2}^{2}+\lambda \left(t\right),s\left(t\right)-\underset{k}{\sum }I_{k}\left(t\right)$$

where α and λ denote the penalty factor and Lagrange multiplier, respectively. $I_{k}^{n+1}$, $f_{k}^{n+1}$ and $\lambda^{n+1}$ are updated as follows:

(4) $${\hat{I}}_{k}^{n+1}\left(f\right)=\frac{\hat{s}\left(f\right)-\sum_{i\neq k}{\hat{I}}_{i}\left(f\right)+\frac{\hat{\lambda }\left(f\right)}{2}}{1+2\alpha {\left(f-f_{k}\right)}^{2}}$$

(5) $$f_{k}^{n+1}=\frac{\int_{0}^{\infty }2\pi f{\left|{\hat{I}}_{k}\left(f\right)\right|}^{2}df}{\int_{0}^{\infty }{\left|{\hat{I}}_{k}\left(f\right)\right|}^{2}df}$$

(6) $${\hat{\lambda }}^{n+1}\left(f\right)={\hat{\lambda }}^{n}\left(f\right)+\varepsilon \left(\hat{s}\left(f\right)-\underset{k}{\sum }{\hat{I}}_{k}^{n+1}\left(f\right)\right)$$

where ε represents the update parameter. In this method, the stop condition is given by:

(7) $$\underset{k}{\sum }\left|\right|{\hat{I}}_{k}^{n+1}-{\hat{I}}_{k}^{n}{\left|\right|}_{2}^{2}/\left|\right|{\hat{I}}_{k}^{n}{\left|\right|}_{2}^{2}<a$$

where a denotes the convergence accuracy. The VMD algorithm can be summarized in: (1) initialize $\left\{{\hat{I}}_{k}^{1}\right\}$,$\;\left\{f_{k}^{1}\right\}$, ${\hat{\lambda }}^{1}$ and n=0; (2) update the values of $\left\{{\hat{I}}_{k}^{n+1}\right\}$,$\;\left\{f_{k}^{n+1}\right\}$ and ${\hat{\lambda }}^{n+1}$ based on Equations (4)–(6); (3) check the covariance condition based on Equation (7), and the details about the VMD algorithm are published in [5].

### 2.2. Permutation Entropy (PE)

PE [15] can not only characterize the randomness of the time series but also detect its dynamic changes. In addition, PE does not consider the amplitude value, but only compares the neighboring values, which makes its operation speed faster. For the given time series $x={\left\{x_{j}\right\}}_{j=1}^{N}$, the PE can be reconstructed as:

(8) $$X_{i}=\left\{x\left(i\right),\;x\left(i+\tau \right),\;\cdots ,x\left(i+\left(m-1\right)\tau \right)\right\},\;i=1,\;2,\;\cdots ,\;N-\left(m-1\right)\tau$$

where m is the embedding dimension, τ is the time delay and i=1,2,⋯,N−(m−1)τ. The elements in $X_{i}$ can be rearranged in increasing order as:

(9) $$x\left(i+\left(j_{1}-1\right)\tau \right)\leq x\left(i+\left(j_{2}-1\right)\tau \right)\leq x\left(i+\left(j_{m}-1\right)\tau \right)$$

If two of the rearranged elements are equal, then,

(10) $$x\left(i+\left(j_{1}-1\right)\tau \right)=x\left(i+\left(j_{2}-1\right)\tau \right)$$

hence, the new order can be denoted as:

(11) $$x\left(i+\left(j_{1}-1\right)\tau \right)\leq x\left(i+\left(j_{2}-1\right)\tau \right)\left(j_{1}\leq j_{2}\right)$$

Therefore, the symbols group can be obtained as:

(12) $$S\left(g\right)=\left(j_{1},j_{2},\cdots ,j_{m}\right)$$

where S(g) represents one of m! symbol sequences in phase space, g=1,2,⋯,k, and k≤m!. If the probability distribution of the symbol sequence is $P_{1},P_{2}\cdots ,P_{k}$, for convergence, the normalized PE is defined as follows:

(13) $$H_{p}\left(m\right)=-{\left(\ln\left(m!\right)\right)}^{-1}\overset{k}{\underset{g=1}{\sum }}P_{g}\ln\left(P_{g}\right)$$

From Equation (13), we can observe that the value of PE ranges from 0 to 1. $H_{p}$ indicates the randomness of time sequence, a larger $H_{p}$ value means higher complexity of the time series; a smaller $H_{p}$ value means lower uncertainty of the time series.

### 2.3. Weighted Permutation Entropy (WPE)

In the PE the neighboring vectors having the same ordinal patterns but with different amplitude values are unreasonably ignored. The WPE [20] has been proposed to take such a situation into account and overcome the PE shortcomings. In the WPE, forgiven embedding dimension m and time delay τ, first, the weight $w_{i}$ of neighbouring vectors $X_{i}$ is calculated as:

(14) $$w_{i}=\overset{m}{\underset{k=1}{\sum }}{\left[x_{j+\left(k-1\right)\tau }-{\bar{X}}_{j}^{m,\tau }\right]}^{2}$$

(15) $${\bar{X}}_{j}^{m,\tau }=\frac{1}{m}\overset{m}{\underset{k=1}{\sum }}x_{j+\left(k-1\right)\tau }$$

Then, the weighted relative frequency is calculated as:

(16) $$p_{w}\left(\pi_{i}^{m,\tau }\right)=\frac{\sum^{\;}j\leq N^{1_{u:\mathrm{type}\left(u\right)=\pi_{i}\left({\bar{X}}_{j}^{m,\tau }\right)}}}{\sum^{\;}j\leq N^{1_{\mathrm{type}\left(u\right)\in \prod^{\;}\left({\bar{X}}_{j}^{m,\tau }\right)w_{j}}}}$$

Finally, the WPE definition is described below:

(17) $$H_{w}\left(m,\tau \right)=-\underset{i:\pi_{i}^{m,\tau }\in \prod^{\;}}{\sum }p_{w}\left(\pi_{i}^{m,\tau }\right)\ln\left(p_{w}\left(\pi_{i}^{m,\tau }\right)\right)$$

### 2.4. Local Tangent Space Alignment (LTSA)

Due to the advantages of insensitivity to parameter selection and fast operation, the local tangent space alignment (LTSA) method has been widely used in dimension reduction in multiple fields [29,30]. The basic idea of the LTSA algorithm is constructing the local tangent space by using the sample neighborhood and mapping the coordinates of the local tangent space corresponding to the global low-dimensional coordinates through the local radiological transformation matrix. Given the data $X=\left\{x_{1},x_{1},\cdots ,x_{m}\right\}\subset R^{M\times N}$, the principle of LTSA can be briefly described as below:

(1) Determine the K nearest neighbors of $x_{i}$ to form the set of $X_{i}$, and centralize ${\hat{X}}_{i}$,
  (18) $$X_{i}=\left[x_{i,1},\;x_{i,2},\cdots ,x_{ik}\right]$$
  (19) $${\hat{X}}_{i}=X_{i}-{\bar{x}}_{i}l_{k}^{T}$$
  where ${\bar{x}}_{i}=\frac{1}{k}\overset{k}{\underset{j=1}{\sum }}x_{ij}$ and $l_{k}$ is a unit vector with dimension K.
(2) Calculate the eigenvalues and eigenvectors of a matrix ${\hat{X}}_{i}$ by singular value decomposition. The eigenvectors corresponding to the first d largest singular values are the tangent space $H_{i}$.
  (20) $$\theta_{ij}=H_{i}^{T}\left(x_{ij}-{\bar{x}}_{i}\right)$$
(3) Construct the transformation matrix $L_{i}=\theta_{i}^{+}$, to retain as much information as possible, and the following conditions must be met,
  (21) $$\min\mu \left(Y\right)=\min\overset{M}{\underset{i=1}{\sum }}\left|Y_{i}\left(I-\frac{1}{k}ll^{T}\right)-L_{i}\theta_{i}\right|$$
  where $\theta_{i}^{+}$ represents the generalized inverse matrix of $\theta_{i}$, $Y_{i}$ represents the set of nearest neighbors of Y after dimension reduction, that is, $Y_{i}=\left(y_{i1},y_{i2},\cdots ,y_{ik}\right)$.
(4) Solve the optimization problem of Equation (21) by calculating the eigenvalues and eigenvectors of the matrix, and then the embedding matrix Y can be obtained. Equation (21) can be equivalent to the following equation:
  (22) $$\min\mu \left(Y\right)=\min\left(YHW\right)=\min tr(YHW^{T}H^{T}Y^{T}),$$
  (23) $$\{\begin{array}{l} H=\left(H_{1},H_{2},\cdots ,H_{M}\right)\;\;\;\;\;\;\;\;\;\;\;\;\;\;\\ W=diag\;\left(W_{1},\;W_{2},\cdots W_{M}\right)\;\;\;\\ W_{i}=\left(I-\frac{1}{k}ll^{T}\right)\left(I-\theta_{i}^{+}\theta_{i}\right)\\ I=YY^{T} \end{array}$$

The low-dimensional embedding matrix Y can be obtained by calculating the eigenvectors corresponding to the second to d-th smallest eigenvalues of the alignment matrix B and it can be calculated as:

(24) $$B=HWW^{T}H^{T}$$

## 3. Proposed Feature Extraction Method Based on Enhanced Variational Mode Decomposition (EVMD), Weighted Permutation Entropy (WPE), and Local Tangent Space Alignment (LTSA)

**Figure 1:**
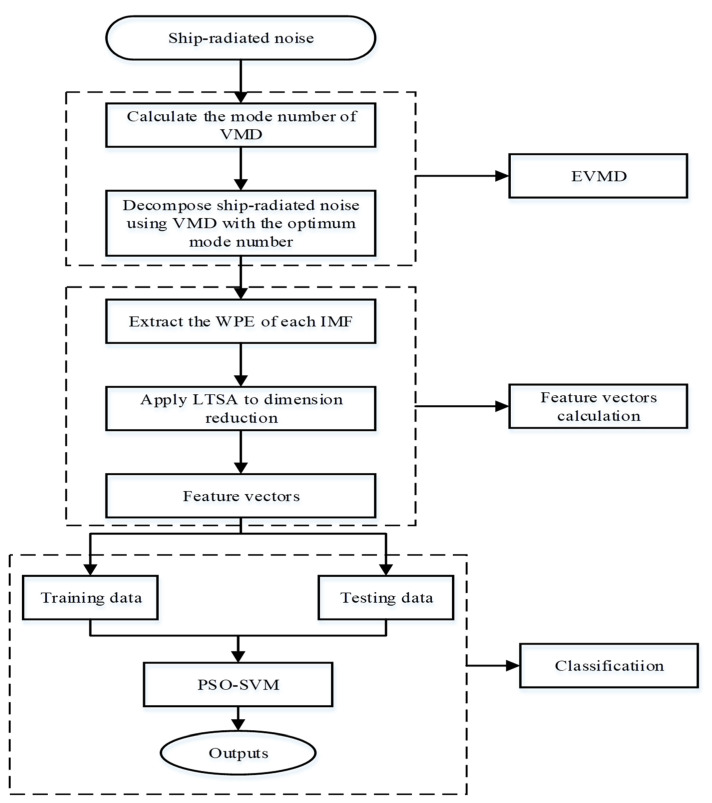
The flowchart of the proposed method.

In Section 2 the details of the basic theories of VMD, WPE, and LTSA are presented. However, the model number of VMD needs to be determined in advance, so this paper proposes the EVMD for SRN signal processing. A multi-stage feature extraction method fully inheriting the advantages of EVMD, WPE, and LTSA is proposed in this paper. The flowchart of the proposed method is shown in Figure 1. The main steps of the proposed method can be summarized as follows:

(1) Based on the center frequency of the intrinsic mode functions, the variational mode decomposition mode number K will be calculated firstly.
(2) The VMD algorithm based on the optimum mode number K obtained in the first step is applied to the decomposition of the SRN signals.
(3) The WPE of each IMF by EVMD is then calculated.
(4) The feature vectors obtained will be randomly divided into two groups, the first group represent the training data used in classification information while the second one is used for evaluating the testing data and measuring its ability for classification.
(5) Definitively, PSO-SVM multiclass classifier uses the analysis data that are recognized and detects the underwater acoustic object.

To extract features of SRN, this paper classifies three types of SRN using a combination of VMD, WPE, LTSA, and PSO-SVM multi-class classifier. In the proposed method, the VMD mode number range is first set according to the EEMD decomposition results, and the variance of the IMFs’ center frequency after each decomposition is calculated. The mode number corresponding to the maximum variance is used as an optimum value for VMD. Then, VMD is performed on three SRN signal types. The WPE value of each IMF for each VMD decomposition is calculated. The high-dimensional features obtained are reduced to be two-dimensional features using LTSA. Finally, the obtained feature vectors are input to the PSO-SVM multi-class classifier to achieve classification and recognition of the samples.

## 4. Simulation Signals Analysis

### 4.1. Analysis of Simulated Signals Based on EVMD

In this paper, as the SRN signals contain the rich line spectral components, the simulated signals composed of the single-frequency components are introduced. In addition, the simulated signals are feasible as long as they satisfy the measurement signal conditions explained in [12], namely, their frequency interval should not be too small (like 1 Hz, 2 Hz, and 3 Hz). Following the same restriction conditions of [12] and verifying the effectiveness and feasibility of the proposed EVMD method. The simulated signals used in this paper are as follows:

(25) $$\{\begin{array}{c} f_{1}\left(t\right)=\cos\left(10\pi t\right)\;\;\;\;\;\;\;\;\;\;\;\;\;\;\;\;\;\;\;\;\;\;\;\;\;\;\\ f_{2}\left(t\right)=\cos\left(60\pi t\right)\;\;\;\;\;\;\;\;\;\;\;\;\;\;\;\;\;\;\;\;\;\;\;\;\;\;\\ f_{3}\left(t\right)=\cos\left(110\pi t\right)\;\;\;\;\;\;\;\;\;\;\;\;\;\;\;\;\;\;\;\;\;\;\;\\ f\left(t\right)=f_{1}\left(t\right)+f_{2}\left(t\right)+f_{3}\left(t\right)+\eta \end{array}$$

where the data length is set to be 5000 with a sampling frequency of 1 kHz and η denotes the Gaussian white noise with CN(0,0.5).

**Figure 2:**
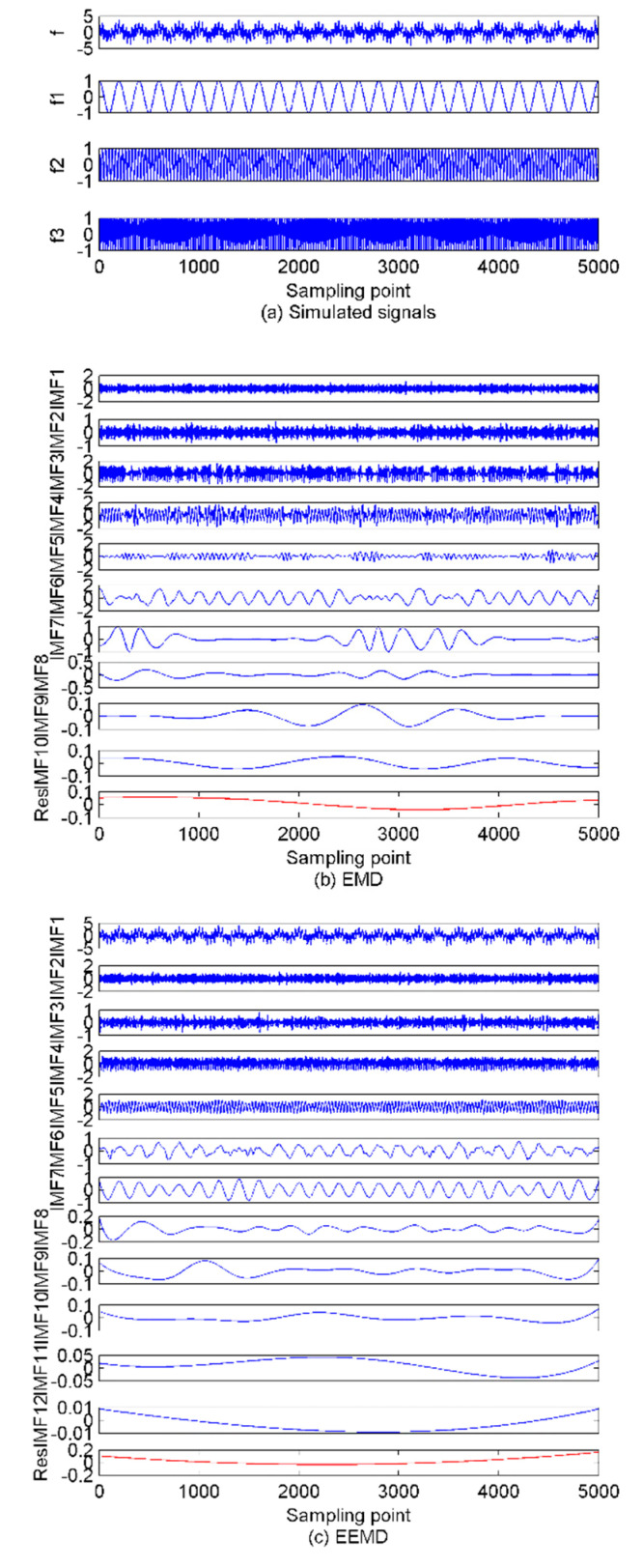
Modeled signals and decomposition results based on empirical mode decomposition (EMD) and ensemble empirical mode decomposition (EEMD) methods. (**a**) the initial modeled signals; (**b**) EMD procedure; (**c**) EEMD procedure.

Following the literature [5], before performing the VMD algorithm, the model number K needs to be adjusted, which serves as a main factor affecting the VMD performance. Other parameters are set as constants, namely the balancing parameter of the data-fidelity constraint is α=2000, the convergence tolerance level is *tol* = 1 × 10^−7^ and the update mode of the center frequency is init=0,1,2 for center frequency iterated with 0, uniform distribution, or randomly. Too-large a K will lead to the occurrence of over-decomposition, which means undesirable spurious components will be generated during the decomposition process; too-small a K will cause under-decomposition, which discards some IMFs carrying useful information during the decomposition process. Hence, a properly chosen K value is crucial to the VMD method. Although the VMD method in [13,14] can successfully decompose the SRN signals to some extent, the method for determining the mode number of VMD has not been reasonably demonstrated, making it unacceptable scientifically. In general, the mode number of VMD will not be greater than the mode number of EMD and EEMD, so conventional EMD and EEMD methods are first employed to analyze the simulated signals above. Figure 2 shows the decomposition results and the time domain waveforms of the simulated signals.

**Figure 3:**
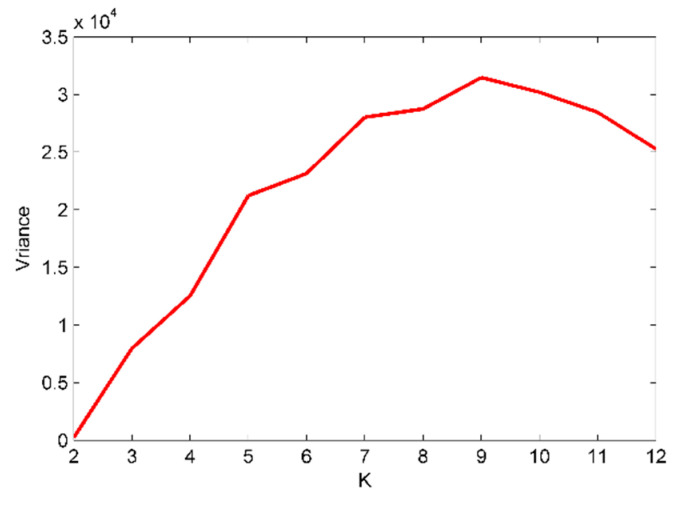
The variance curve of intrinsic mode functions (IMFs) center frequency with mode number K after simulated signals decomposition using variational mode decomposition (VMD).

Figure 2, show modeled indicator decomposed by using the EMD, one remaining part is obtained in addition to 10 IMFs. While in the EEMD decomposed method there are one remaining part and 10 IMFs, and they are separated. Hence, based on setting the variables for the mode number range to be (2~12), and following calculation of every decomposition IMFs’ center frequency, the good mode number K has the maximum variance which maximizes the IMFs center frequency difference. Figure 3, shows the IMFs’ center frequency at different mode number K when the VMD decomposing method is used, and Figure 3 shows that K=9 is the best choice.

**Figure 4:**
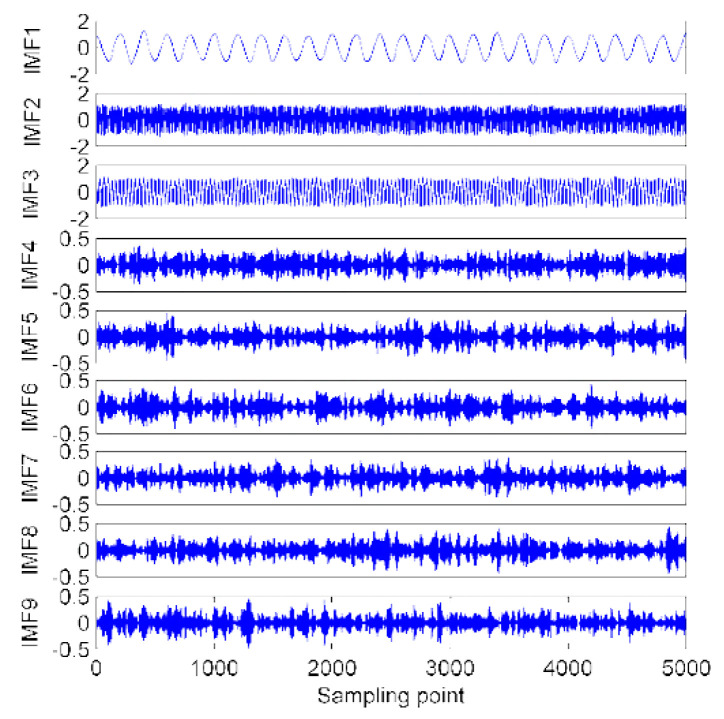
The EVMD decomposition results for the simulated signals.

When the mode number K is set to be more than the good, estimated value K=9, the variance begins to reduce and that shows irrelevant variation among the IMFs’ center frequency and the existence of the done decomposition. Figure 4, shows the decomposition results of the modeled signals based on the EVMD procedure. The IMFs’ center frequency distribution at different mode numbers K are listed in Table 1. Table 1 shows how components are unseparated for K=(2~6), and for K≥7 the modeled signal is separated. For K=(10~12) further false components are created to recognize the split of the modeled signals.

Based on the analysis above, the proposed EVMD method using the variance of the IMFs’ center frequency is feasible in calculating the mode number of VMD. To further verify this method in the VMD algorithm, the VMD method in [4,20] is also introduced for comparison. The correlation coefficients between the corresponding IMF and simulated signals are calculated and the results are listed in Table 2. As shown in Table 2, the corresponding three components of the proposed EVMD have the highest correlation coefficients with the simulated signals, and its decomposition performance is significantly better than the conventional methods of the EMD, EEMD, and VMD. This confirms the validity and feasibility of the method proposed in this paper for determining the VMD mode number.

### 4.2. Analysis of the Properties Concerning Weighted Permutation Entropy (WPE) and Permutation Entropy (PE)

**Figure 5:**
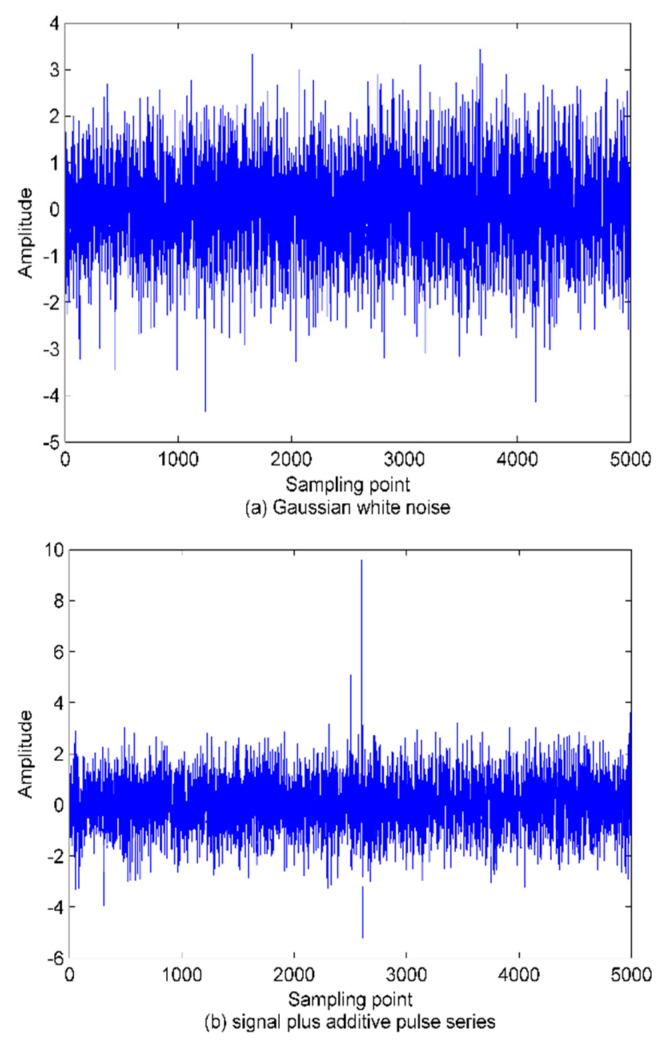
The time domain diagrams of Gaussian white noise and the signal plus additive pulse series.

**Figure 6:**
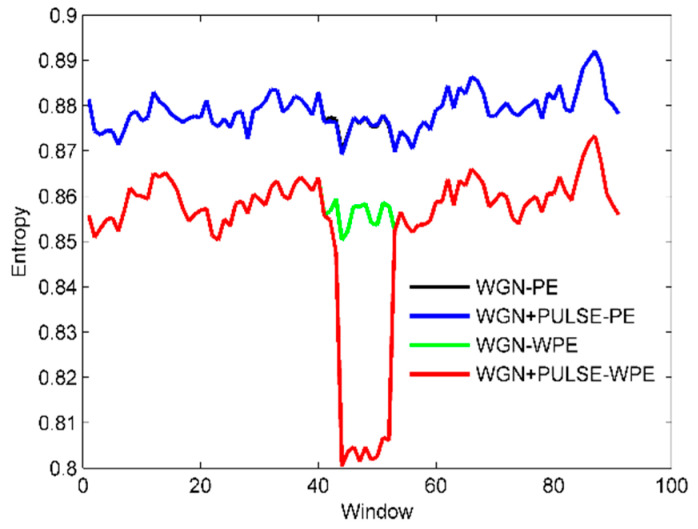
The curve of PE and WPE with window.

According to the basic principles of WPE and PE explained in Section 2, when the signal is mutated, it is difficult for PE to detect this state, while WPE should be more sensitive to this mutation. To validate the conjecture, we generate a standard Gaussian white noise series with a length of 5000. As the pulse series means a larger fluctuation, we add the pulse series to these Gaussian white noise series. As in [31], the time delay and the embedding dimension are set as 1 and 6, respectively. The PE and WPE are calculated using a window function with a length of 500 and a sliding step of 50. The time-domain waveforms of the Gaussian white noise series and the signal plus additive pulse series are shown in Figure 5. The results of the calculated entropy are shown in Figure 6.

As shown in Figure 6, the WPE values of two signals of the SRN are smaller than the corresponding PE ones. This indicates how Gaussian white noise has a higher complexity and contains more information. There is no difference in the PE of the two signals in the pulse region, which means the inability of PE to distinguish between these two signals. This can be attributed to the neglect of the amplitude difference between neighboring vectors having the same ordinal patterns in PE calculation. As a result, PE performs poorly in effectively detecting fluctuations caused by noise. In contrast, the WPE value of the signal after superimposed pulses decreases significantly in the pulse region, indicating that the WPE can effectively detect the amplitude-encoded information contained in the signal due to the introduction of weights, which outperforms the PE in noise detection and fluctuation observation.

**Figure 7:**
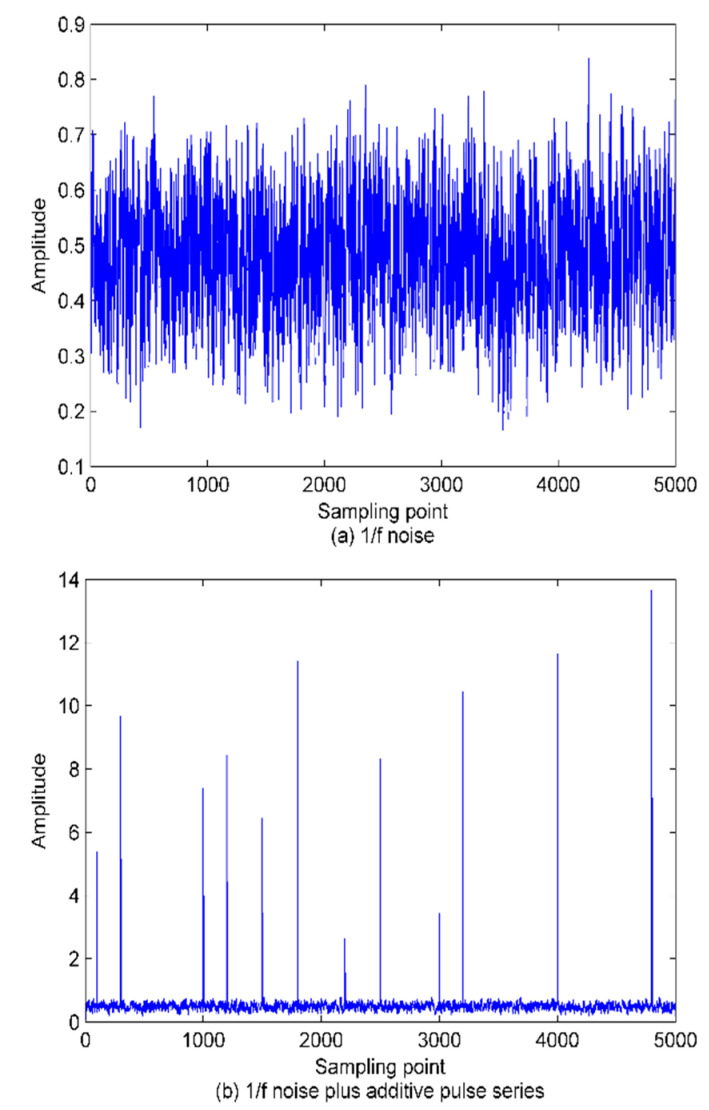
The time domain waveforms for; (**a**) 1/f noise (**b**) the signal plus additive pulse series.

**Figure 8:**
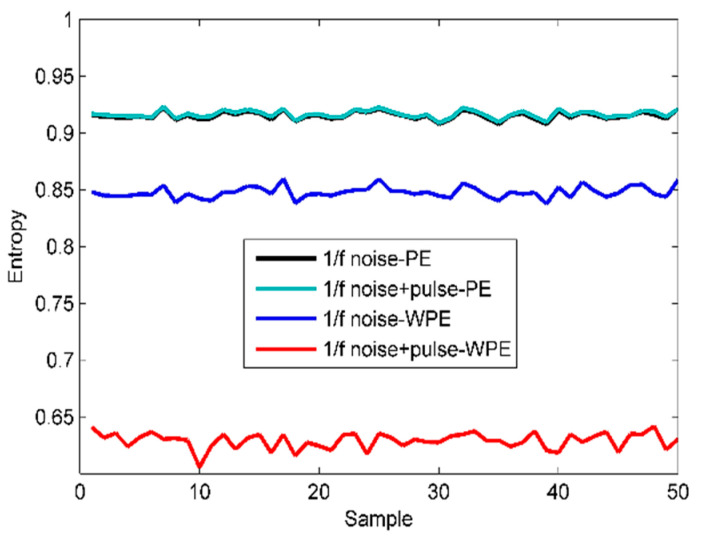
The scatter plots of calculation results for PE and WPE.

For further comparison between the performance of the PE and WPE, 50 1/f noise samples with a length of 500 are generated and the pulse series is superimposed on the raw 1/f noise series. For a fair comparison, PE and WPE calculations concern the two signals. The time-domain waveforms of the analyzed signals and the scatter plots of the calculation results are given in Figure 7 and Figure 8, respectively.

As shown in the results, thanks to the good anti-noise ability of WPE, making the estimated WPE value of the two signals is lower than the PE value. Also, sudden change detection is a hard task in the PE method as it neglects the amplitude information. In contrast, except for the significant difference in WPE values between the two signals, the WPE fluctuation trends are more dramatic than PE. Larger fluctuation means stronger discrimination ability. In short, the analysis of experimental results proves the advantages of WPE over PE.

## 5. Feature Extraction of Ship-Radiated Noise Based on the Proposed Method

### 5.1. Parameter Selection

The WPE calculation is dependent on the time delay τ and embedding dimension m. If m is too small, the reconstructed sequence will contain less state information, and the WPE algorithm cannot adequately detect the dynamic change of the time series. However, larger m indicates that the time series is homogenized by the reconstructed phase space and cannot detect the time series slight change. Also, if τ is too small, a strong correlation will occur between different delay vector elements, resulting in information redundancy. The phase space trajectory cannot be fully expanded when τ is too large. Therefore, such parameters should be adjusted first before WPE calculation.

**Figure 9:**
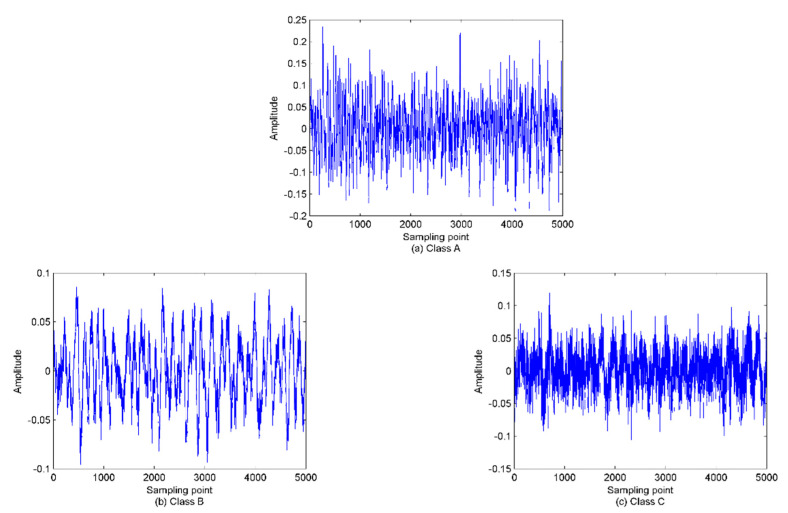
The time-domain waveforms for the three types of normalized ship−radiated noise (SRN).

To study the influence of m and τ on PE and WPE, the three types of SRN are randomly selected from the data set used in [32]. The samples were recorded on the Atlantic coast in north-western Spain $\left(42^{{}^{\circ}}\;14^{\prime }\;N,\;008^{{}^{\circ}}\;43.4^{\prime }\;W\right)$ at a depth of 10 m. The sampling frequency is 52.734 kHz and the data length is set to be 5000. The three types of SRN signals are named class A, B, and C, respectively. Figure 9 shows the time-domain waveforms of the normalized signals.

**Figure 10:**
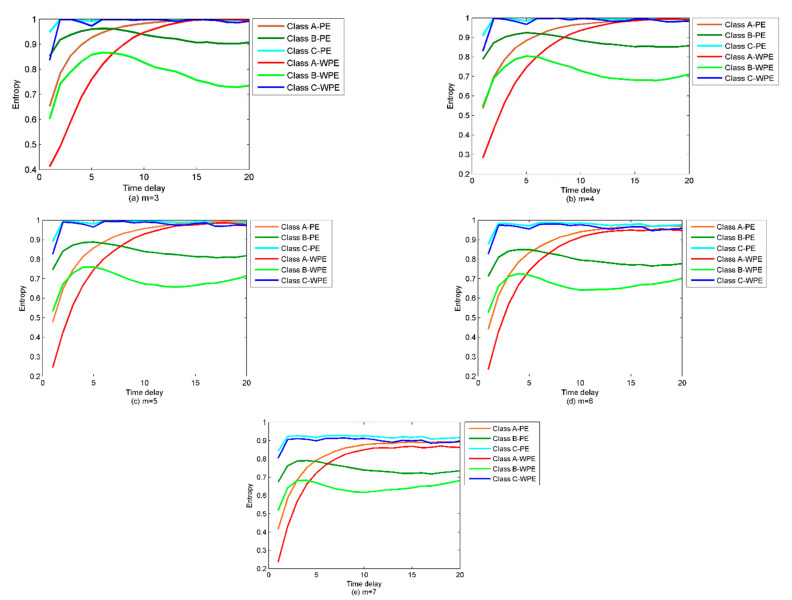
PE, WPE comparison of the three types of SRN under different embedding dimensions.

As in [30], the PE and WPE of the three types of SRN signals are calculated under the condition m = 3, 4, 5, 6, 7, and the time delay ranges from 1 to 20. The results are shown in Figure 10. As seen in Figure 10, the class C SRN signal has the maximum entropy value and thus the highest complexity.

**Figure 11:**
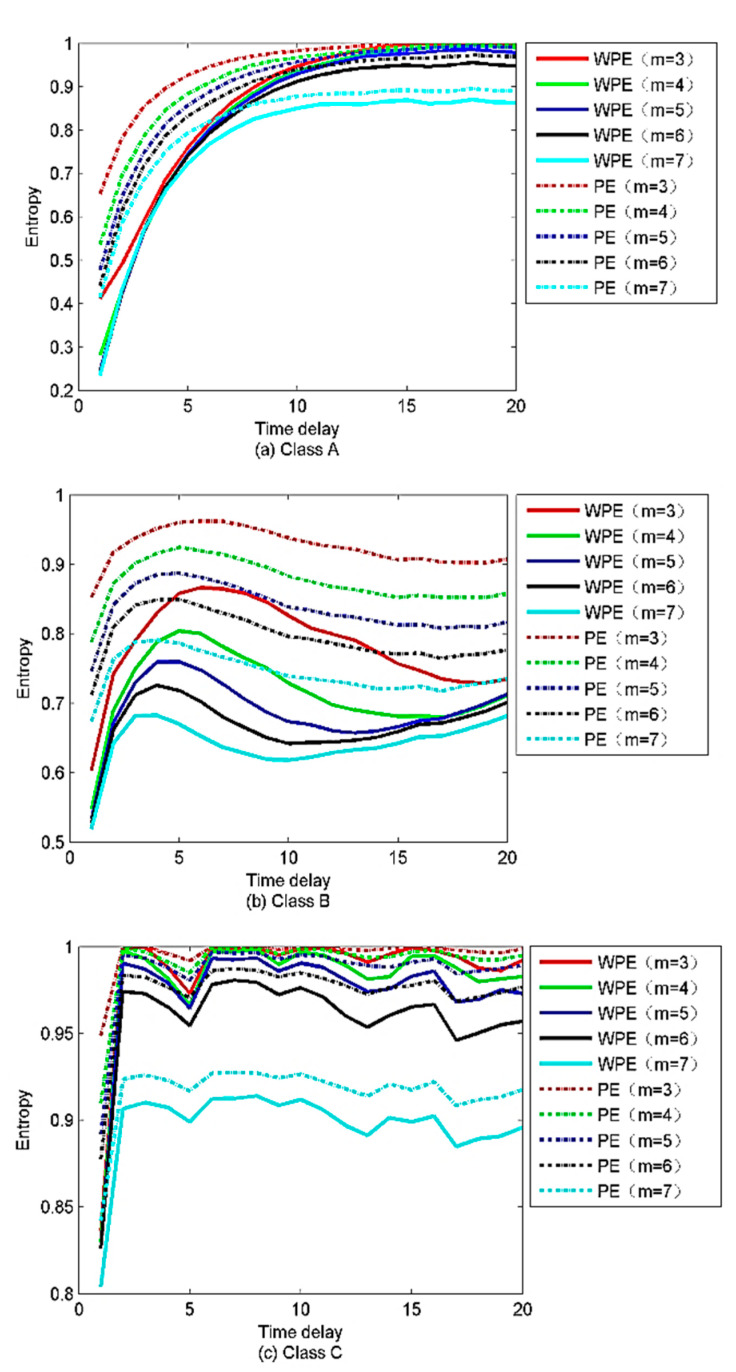
PE and WPE of the signals varying with the time delay.

Thanks to the excellent anti-noise performance of WPE, the WPE value of the same time series is less than the PE. WPE is more sensitive to the time delay compared to the PE, as the PE fluctuates subtly with the time delay increasing. The WPE and PE begin to separate when *m* = 6. However, when m is increased to 7, the computational complexity will be increased without improving the accuracy of calculation results. Based on the results obtained in Figure 10, a significant difference occurs between the WPE and PE when the time delay is equal to 1. Therefore, in the proposed method when calculating the WPE and PE, the delay and embedding dimensions are set to be 1 and 6, respectively. These parameters are consistent with the recommendations given by [15,31]. The PE and WPE of the signals versus the time delay are shown in Figure 11. As shown in Figure 11, the time delay has a greater influence on PE and WPE in some ranges, while less in others as the embedding dimension increases. Compared to PE, the trends of WPE fluctuate relatively sharply, as the WPE is more sensitive to the pattern extracted from signals containing amplitude information.

### 5.2. Decomposition of Ship-Radiated Noise Using VMD

**Figure 12:**
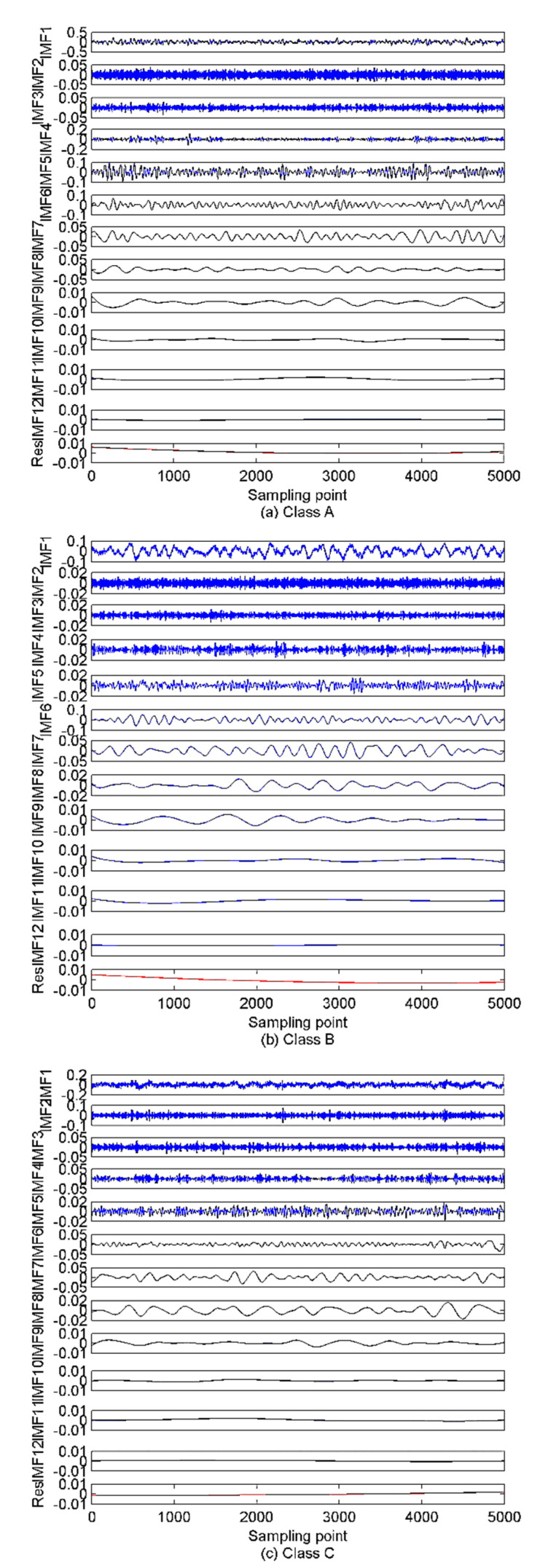
The EEMD results for SRN signals.

**Figure 13:**
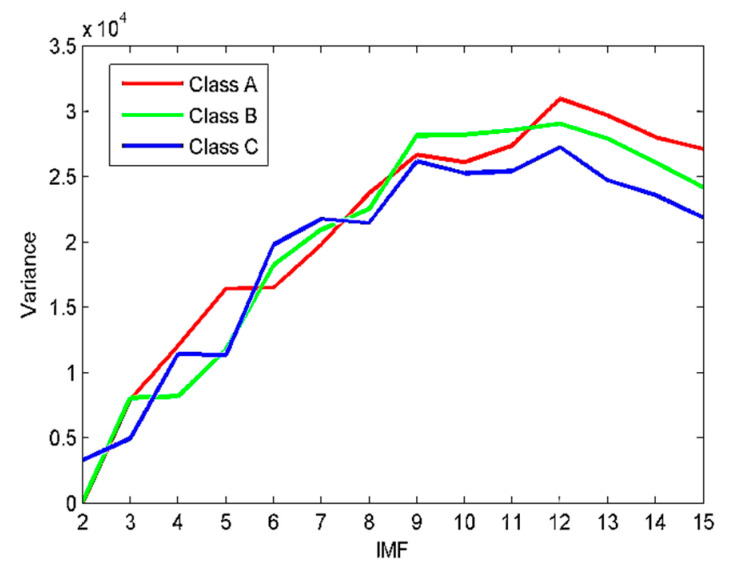
The results of variance analysis for the SRN signals.

**Figure 14:**
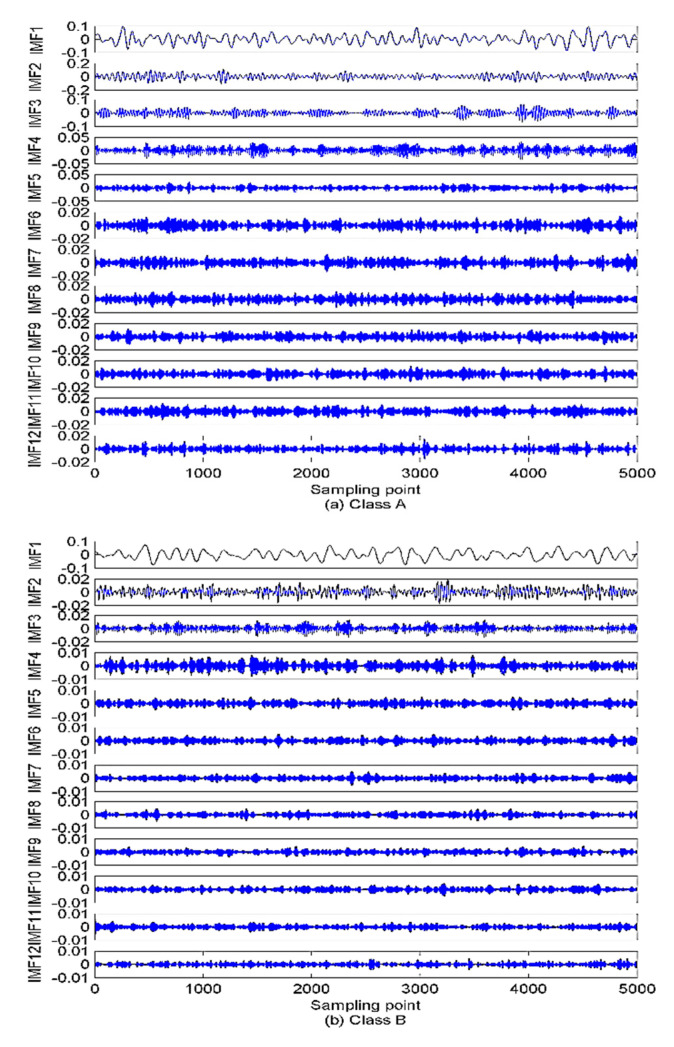

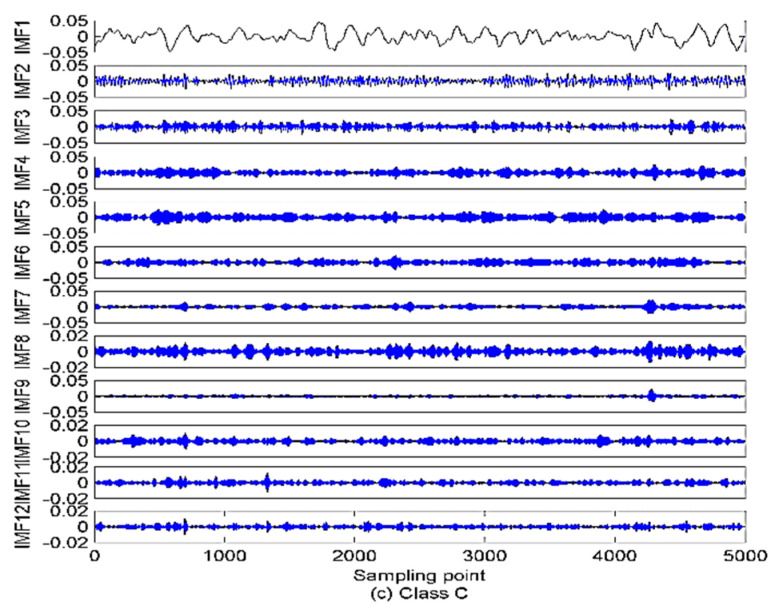
The EVMD results for SRN signals.

As described in Section 3, to calculate the VMD model number, the EEMD algorithm is first employed to decompose the SRN signals. The decomposition results are presented in Figure 12. Figure 12 shows that 12 IMFs and one residual component are obtained after the EEMD of each type of SRN signal. For the sake of observation, K is set as (2~15) to calculate the variance of the IMFs’ center frequency. The results are given in Figure 13. It can be observed from Figure 13 that the optimal K is 12, and when K is higher than 12, the variance starts to decrease sharply, implying the occurrence of over-decomposition. The decomposition results by EVMD are given in Figure 14.

### 5.3. Classification of Ship-Radiated Noise (SRN)

**Figure 15:**
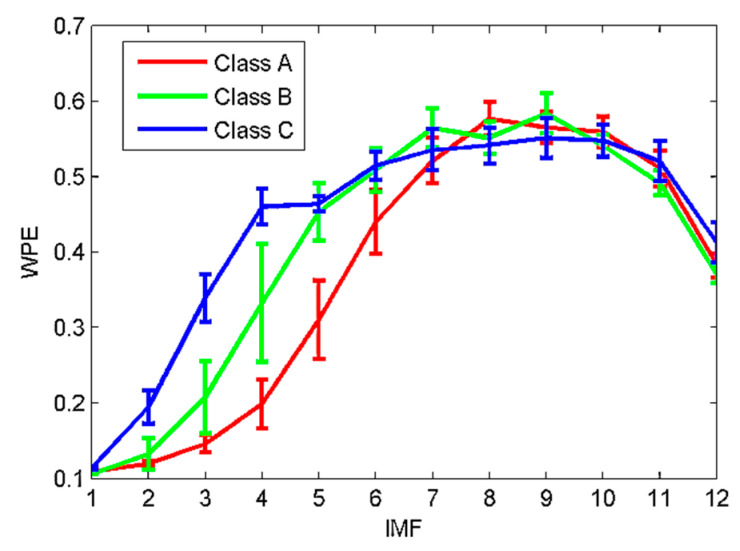
The mean and standard deviation of WPE.

In this section, the proposed method is applied to the SRN samples classification. 100 samples are randomly selected from each type of SRN sample and thus a total of 300 samples can be obtained. The EVMD is first performed to decompose these samples and the WPE of each IMF is calculated. Figure 15 shows the WPE mean and standard deviation.

Figure 15 shows, when the mode is IMF3 or IMF4, that the three types of samples can be distinguished, and the WPE values of class C samples are higher than that of the other two classes. This can be due to the dynamic behavior changes at different signals. Hence, WPE can effectively reflect the dynamic changes of the time series. However, when the mode is IMF2, the class A and B samples cannot be identified; when IMF5, only class A can be identified. In other modes, all three types of sample are indistinguishable. Therefore, not every IMF can fully characterize the raw SRN signal after the proposed EVMD decomposition. The results can be attributed to two points.

**Figure 16:**
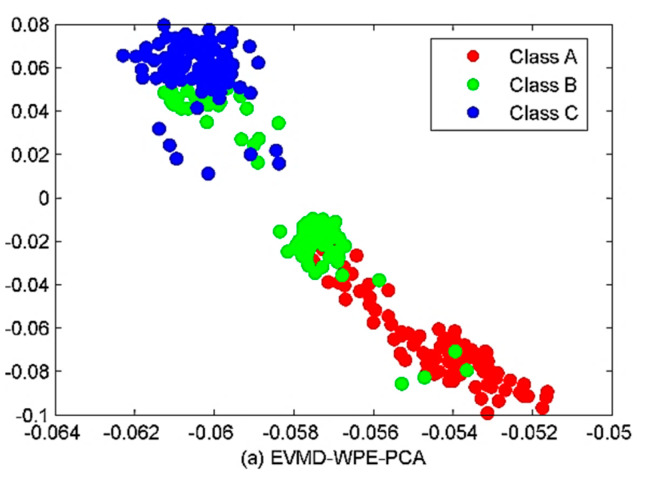

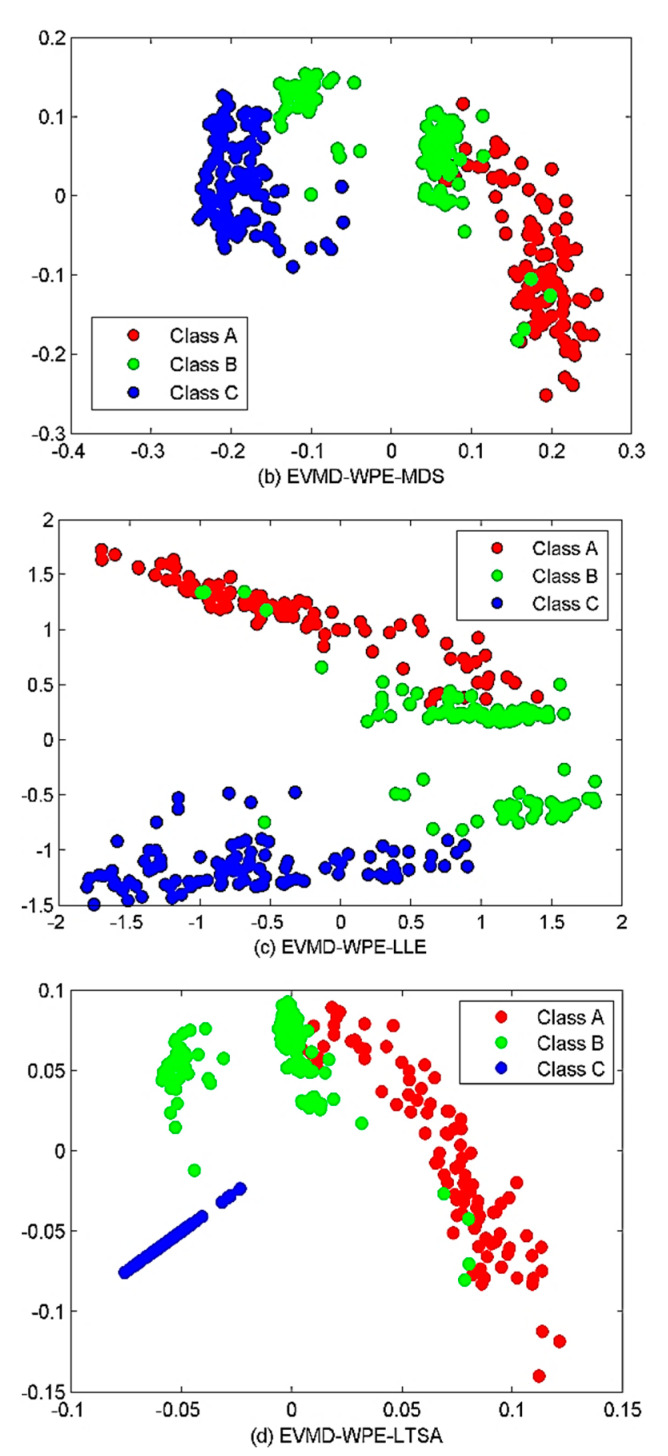
The clustering results of the four algorithms.

**Figure 17:**
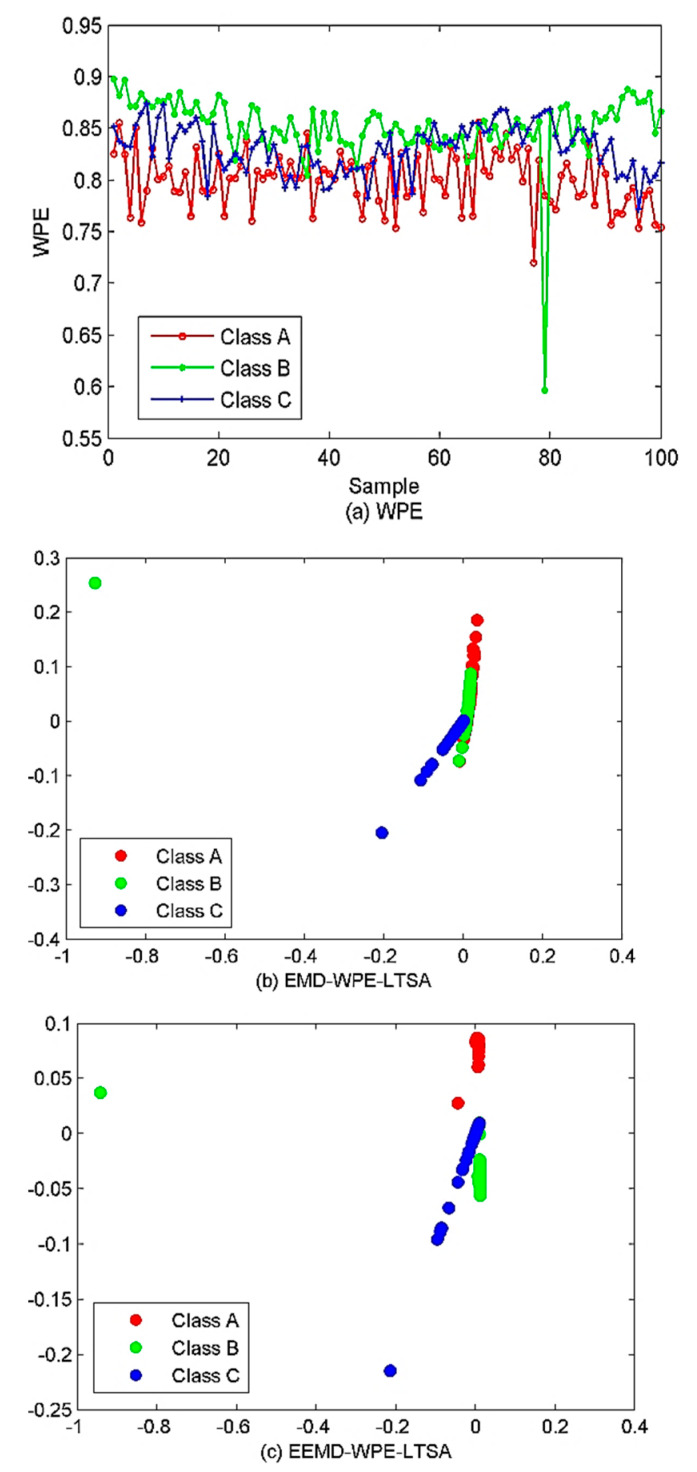
The scatter plots of the three algorithms.

Firstly, due to the pollution of marine environmental noise, some IMFs belong to noise or noise-dominant components. Secondly, the occurrence of over-decomposition in the VMD algorithm allows different IMFs to share the same spectrum information. In this way, the dimension reduction algorithms are introduced to avoid dimensional disasters, thus preparing for the classification below. Next, the PCA, MDS, LLE, and LTSA methods are utilized for the low-dimensional feature extraction. The number of neighboring points and the target dimension are set as 15 and 2, respectively. The results are given in Figure 16. As shown in Figure 16, overall, most of the three types of samples can be distinguished by the four algorithms despite the partially overlapping samples between classes A and B. From a qualitative point of view, the combined EVMD-WPE-PCA algorithm has shown the worst performance with a small number of samples crossing between class A and B, and more samples overlapping between class B and C, while the performance of EVMD-WPE-MDS and EVMD-WPE-LLE has been significantly improved compared to EVMD-WPE-PCA with only a few samples of class A and B crossed. Despite the good performance of EVMD-WPE-PCA, EVMD-WPE-MDS, and EVMD-WPE-LLE in roughly identifying the three types of samples, the degree of clustering within the class is still small, especially for class B. In contrast, the proposed combined EVMD-WPE-LTSA method has the best clustering performance. The three kinds of SRN sample can be separated well, and the samples of classes B and C are better clustered. For a fair comparison, the scatter plots of WPE, EMD-WPE-LTSA and EEMD-WPE-LTSA are represented in Figure 17.

**Figure 18:**
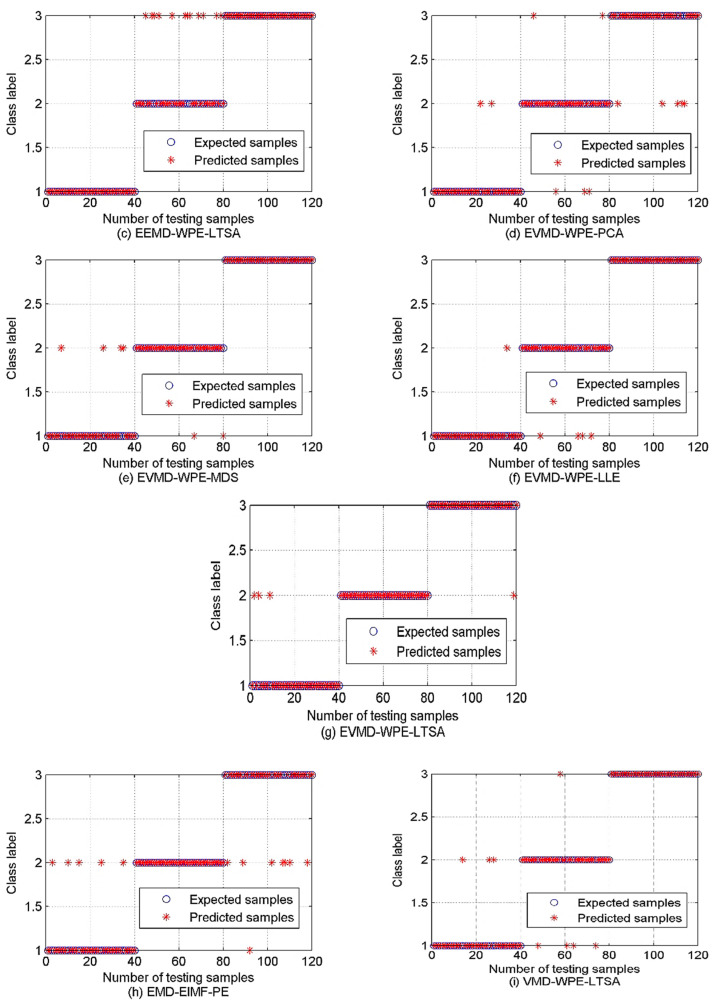
The outputs of classification under different algorithms.

As shown in Figure 17a, affected by the oceanic environment and the ambient noise, we cannot recognize the majority of the three forms of samples as they will be opposed together. Hence in such a case, the algorithms of the decomposition should be used. For the EMD-WPE-LTSA shown in Figure 16b, a large proportion of samples of classes A and B are overlapped, which cannot be separated well. The samples in the C category are also intersected with the others. Hence, recognition of the three samples becomes difficult. While concerning the EEMD-WPE-LTSA, the samples in categories A and B can be completely separated, but the degree of separation between categories B and C is still small. From a qualitative point of view, there is no significant difference between EEMD-WPE-LTSA and EVMD-WPE-LTSA, and EEMD-WPE-LTSA even looks more clustered within the class, but we cannot conclude on the merits of both algorithms. Data visualization is only a qualitative tool rather than a quantitative one. In this situation, the PSO-SVM multi-class classifier is introduced to compare the algorithms above more accurately. Next, 60 samples are randomly selected from each class to be used as a training classifier and the remaining samples are left to be used in testing the performance. Also, both EMD-EIMF-PE [21] and VMD-WPE-LTSA (the mode number of VMD is set as [4,20]) methods are considered. The classification outputs are shown in Figure 18. Table 3 lists the classification accuracy and computational time for SRN feature extraction under different algorithms.

As shown in Figure 18 and Table 3, the direct calculation of the WPE for the samples fails to achieve the SRN samples identification as it has only a 62.5% recognition rate and that is far from the classification standard. The multistage classification techniques based on the proposed EVMD have the highest classification accuracy of 95.8333% and 96.6667% in EVMD-WPE-LLE and EVMD-WPE-LTSA, respectively. The proposed multistage classification techniques based on EVMD outperform the other conventional algorithms with the additional computational time cost. In general, computational complexity and classification accuracy are determined by the configuration of the hardware and algorithm design.

## 6. Conclusions

A novel multi-stage feature extraction method for underwater acoustic signals is proposed in this paper based on combining the new EVMD method with WPE and LTSA. The main innovations and contributions of this work can be summarized as follows:

(1) The proposed EVMD method mode number was calculated based on the variance of IMFs’ center frequency.
(2) WPE and LTSA were combined with the new EVMD and applied to underwater SRN signals for the first time.
(3) The proposed EVMD-WPE-LTSA was successfully applied, and extracted the features of the underwater acoustic signals. Compared with other algorithms, EVMD-WPE-LTSA had a higher recognition rate and better classification performance at the cost of computational time.

The EVMD algorithm proposed in this paper to overcome the shortage of the VMD performed accurately in the field of underwater acoustic communication. Nevertheless, this paper only addresses the VMD mode number. In future, the optimization between both the model number and the quadratic penalty term will be considered to obtain high decomposition accuracy for the EVMD. Also, we will try to reduce the computation complexity.

## Figures and Tables

**Table 1:** The mode number corresponding to the distribution of IMF’s center frequency by VMD.

K	Center Frequency/Hz											
2	7.79	52.50										
3	7.77	52.50	310.22									
4	7.73	52.51	193.88	358.92								
5	7.71	52.52	178.13	306.10	429.27							
6	7.63	52.53	132.93	231.63	325.67	438.33						
7	5.04	55.14	30.02	178.15	281.03	365.89	468.10					
8	5.04	55.12	30.02	150.48	230.17	304.96	377.06	470.06				
9	5.04	55.11	30.02	133.53	202.41	280.99	349.96	413.83	476.09			
10	5.04	55.09	30.01	122.45	181.76	240.15	300.08	359.29	418.56	477.08		
11	5.03	55.09	30.01	118.11	173.42	227.27	276.65	321.81	369.88	424.40	478.29	
12	5.03	55.05	30.01	99.61	146.79	192.70	237.79	283.66	325.96	372.20	425.66	468.56

**Table 2:** Correlation coefficients between corresponding IMF with simulated signals.

	EMD	EEMD	VMD	EVMD
*f*_1_	IMF6: 0.9244	IMF7: 0.9665	IMF1: 0.9938	IMF1: 0.9944
*f*_2_	IMF4: 0.8959	IMF5: 0.9806	IMF3: 0.9914	IMF3: 0.9925
*f*_3_	IMF3: 0.8875	IMF4: 0.9460	IMF2: 0.9872	IMF2: 0.9880

**Table 3:** The classification accuracy and computational complexity under different algorithms.

Method	NUMBER OF MISCLASSIFIED SAMPLES			ACCURACY RATE (%)	COMPUTATIONAL TIMES (SECOND)
	CLASS A	CLASS B	CLASS C		
WPE	11	7	27	62.5	787.35041
EMD-WPE-LTSA	11	9	2	81.6667	6956.81681
EEMD-WPE-LTSA	0	12	0	90	23,643.2661
EVMD-WPE-PCA	2	5	5	90	22,254.0133
EVMD-WPE-MDS	4	2	0	95	22,255.2634
EVMD-WPE-LLE	1	4	0	95.8333	22,255.0276
EVMD-WPE-LTSA	3	0	1	96.6667	22,255.0041
EMD-EIMF-PE	5	0	8	89.1667	1342.94618
VMD-WPE-LTSA	3	5	0	93.3333	12,404.5999

## Data Availability

The data used to support the findings of this study are available from the corresponding author upon request.
